# Leveraging the social networks of informal healthcare providers for universal health coverage: insights from the Indian Sundarbans

**DOI:** 10.1093/heapol/czae060

**Published:** 2024-11-18

**Authors:** Rittika Brahmachari, Manasee Mishra, George Gotsadze, Sabyasachi Mandal

**Affiliations:** Children International, 2000 E Red Bridge Rd, Kansas City, MO 64131, United States; Parul University, P.O Limda, Ta.Waghodia, Vadodara, Gujarat 391760, India; Curatio International Foundation, 3 Kavsadze str, Tbilisi, Georgia 0179, Georgia; School of Natural Science and Medicine, Ilia State University, 3/5 K. Cholokashvili Ave, Tbilisi, Georgia 0162, Georgia; Tata Steel Foundation, 3,E-Road, Northern town, Bistupur, Jamshedpur, Jharkhand 831001, India

**Keywords:** Informal healthcare providers, intersectoral collaboration, universal health coverage, qualitative social network analysis, health systems

## Abstract

India’s healthcare landscape is characterized by a multitude of public and private healthcare providers, yet its health systems remain weak in many areas. Informal healthcare providers (IHPs) bridge this gap, particularly in rural India, and are deeply embedded within local communities. While their importance is widely recognized, there is a knowledge gap regarding the specifics of their social networks with actors in health systems. The aim of this study was to map the social networks of IHPs to elucidate the type and nature of their relationships, in order to explore opportunities for intersectoral collaboration to achieve universal health coverage (UHC). We have adopted the social network analysis (SNA) approach using qualitative ego-network methodology to evaluate the types and strengths of ties in the Indian Sundarbans. A total of 34 IHPs participated in the study. Qualitative data were analysed using NVivo10 and Kumu.io was used to visualize the social networks. Results show that the 34 IHPs had a total of 1362 ties with diverse actors, spanning the government, private sector and community. The majority of the ties were strong, with various motivating factors underpinning the relationships. Most of these ties were active and have continued for over a decade. The robust presence of IHPs in the Indian Sundarbans is attributable to the numerous, strong and often mutually beneficial ties. The findings suggest a need to reconsider the engagement of IHPs within formal health systems. Rather than isolation, a nuanced approach is required based on intersectoral collaboration capitalizing on these social ties with other actors to achieve UHC in impoverished and underserved regions globally.

Key messages

- Healthcare is provided by informal healthcare providers (IHPs) in many poor and underserved areas of the world where formal health systems are deficient. IHPs have strong, active, numerous and mutually beneficial ties with wide-ranging private, government and community actors.
- Such ties help IHPs in the continuous acquisition of knowledge and skills to provide healthcare in a geographically challenging region of India. The ties are also instrumental in safeguarding their position in local communities.
- The social ties of IHPs could be leveraged to achieve universal health coverage through multiactor involvement in low- and middle-income countries that struggle to provide qualified human resources for health service delivery in poor and underserved areas.

## Introduction

India and many low- and middle-income countries face a shortage of trained clinical staff, mostly in rural areas (Naicker *et al*., 2010; Sharma, 2015; Drennan and Ross, 2019; Karan *et al*., 2021; Nair *et al*., 2022). Geographic Information System mapping of health centres by Chandra and Bhattacharya (2019) revealed that a person needs to travel 5–10 km to consult a qualified doctor. Another survey of the health workforce in India by Karan *et al*. (2021), found that 50% of staff are not adequately qualified. Further, out of the qualified workers, 77% reside in urban areas, serving only 31% of the country’s urban population. More than 8% of the country’s 25 300 primary health centres lack a doctor (Sharma, 2015). This shortage of healthcare workers and accessibility issues, along with a poor regulatory framework (Kanjilal *et al*., 2010; Sudhinaraset *et al*., 2013), drive the need for informal healthcare providers (IHPs) (Bhuiya, 2009; Mahmood *et al*., 2010). IHPs include traditional practitioners and chemists, among others, who practice medicine without any formal medical qualifications or accreditation (Sudhinaraset *et al*., 2013; Gautham *et al*., 2014; Das *et al*., 2016). IHPs comprise a significant part of the private health sector not only in India but also in South Asia and Sub-Saharan Africa. The primary reasons for the dependency of rural communities on IHPs include proximity, affordability, round-the-clock availability and trust (Kanjilal *et al*., 2007; Bhuiya, 2009; Mahmood *et al*., 2010; Gautham *et al*., 2011; Sudhinaraset *et al*., 2013; Khare *et al*., 2022).

While IHPs play a significant role in providing healthcare, particularly in underserved rural areas, their presence also raises concerns regarding patient safety, quality of care and adherence to medical protocols. Studies have highlighted instances of misdiagnosis, inappropriate treatment and the over prescription of antibiotics, which leads to potential risks to patient health due to lack of training and oversight (Nair *et al.*, 2019b; Gautham *et al*., 2021). To address the concerns raised over the last few years, several attempts have been made to expand the capacity of IHPs in Asian countries such as India, Bangladesh and China, as they are often the first point of contact for rural communities (Kanjilal *et al*., 2007; Sudhinaraset *et al*., 2013; Islam *et al*., 2014; Das *et al*., 2016; Gautham *et al*., 2022). It has been shown that training improves IHP knowledge and their continued integration into health systems, for preventive health, can help achieve universal health coverage (UHC) (Das *et al*., 2016; Mahapatra *et al*., 2021; Gautham *et al*., 2022; Kumah, 2022; Nayak *et al*., 2022; Sun *et al*., 2022; Thapa *et al*., 2023). Though several studies have demonstrated that training IHPs results in improved rates of correct case management, it did not decrease the inappropriate use of medications such as unnecessary medicines or antibiotics (Das *et al*., 2016; Nair, et al., 2019a; Sun *et al*., 2022). Importantly, the training did not lead to an increase in medical guideline violations or a decline in clinical practice standards, which were concerns raised by representatives of the Indian Medical Association (Gautham *et al*., 2021). Furthermore, existing research has explored the relationships between IHPs and broader health systems revealing complex collaboration patterns, referral networks and resource sharing dynamics (Sudhinaraset *et al*., 2013; Gautham *et al*., 2021). Additionally, Rahman-Shepherd *et al.* (2021) points out that a conflict of interest exists between formal and informal healthcare providers due to hidden financial relationships. The referral networks of IHPs with private doctors and pharmaceutical companies as sources of knowledge regarding new drugs and antibiotics has also been highlighted (Nair *et al.*, 2019b). Mapping of the social network of IHPs with health systems and other actors can contribute to a greater understanding of their relationship and how networks function (Sudhinaraset *et al*., 2013). However, there is little evidence on the overall picture of how IHPs are connected with other actors in health systems. Such research could contribute to achieving UHC by leveraging intersectoral collaboration as it helps to understand how IHPs are connected with different actors and the reasons that bind the relationships. According to the World Health Organization (WHO), as described by Nutbeam (1994), intersectoral collaboration is defined as actions taken by other sectors, along with the health sector, to improve health outcomes sustainably and effectively. Several studies have underscored the potential of collaboration between formal and informal health sectors to improve access to health services (Mangham-Jefferies *et al*., 2014; Shah *et al.*, 2011). Intersectoral collaboration facilitates the integration of IHPs into the broader health systems, leveraging their proximity to communities and cultural relevance to enhance service delivery (Kumah, 2022). Partnership is required between government, and non-government and community-based organizations (CBOs) (Nutbeam, 1994). However, regulatory barriers, lack of standardized training and coordination issues pose significant obstacles to seamless intersectoral collaboration. This research aims to contribute to existing knowledge by providing a comprehensive understanding of the various actors in the social network of IHPs and how such ties can be leveraged to enhance healthcare access through intersectoral collaboration in underserved areas.

## Methods

Social network analysis (SNA) is a methodology that allows an understanding of different actors, the relationships between them and the flow of resources (Wasserman and Faust, 1994; Scott, 2012). It is rarely used to understand ties between health system actors and the associated implications for health service delivery in low- and middle-income countries (Sabot *et al*., 2017; Hu *et al.*, 2021). Blanchet and James (2012) advocate using SNA because it allows new insights into the structure of health systems and facilitates understanding of the role of different actors in it. Using qualitative SNA (Edwards, 2010) with ‘qualitative ego-network’ methodology (Wasserman and Faust, 1994; Emmel and Clark, 2009; Chamberlain, 2006), our study investigates the types and strength of the ties and changes in the network over time.

### Study setting

This study was conducted in the Indian Sundarbans, a geo-climatically vulnerable region at the confluence of the river Ganges with the Bay of Bengal. It encompasses 106 islands, 54 of which are inhabited and face threats such as cyclones and floods due to the deltaic topography. Geographical barriers hinder healthcare access. Hours of travel across land and rivers are often required to reach formal healthcare facilities (Kanjilal *et al*., 2010). Weak infrastructure and physician shortages make village-level primary health centres inaccessible. Despite the government employing frontline health workers such as Accredited Social Health Activists (ASHA), 16% of positions remain vacant (Kanjilal *et al*., 2010). Consequently, unqualified IHPs dominate outpatient care at 62%, surpassing that of rural West Bengal (the parent state). They mostly practice allopathic medicine without a formal qualification (Kanjilal *et al*., 2010).

### Sampling

Kanjilal *et al*. (2010) identified six administrative blocks in the Indian Sundarbans as ‘priority’ blocks in terms of health service availability and physical inaccessibility. The study was conducted in one block of South 24 Parganas district with a population of 331 823 according to the 2011 census. This region has one rural hospital, three primary health centres and several Non Governmental Organisation (NGOs)/private nursing homes. According to census data, several villages in the block lack medical facilities and the main government medical facility in the block has 30 beds. Kanjilal *et al*. (2010) highlighted inadequacies in primary health centres, poor infrastructure of Block Primary Health Centres (BPHCs) and a shortage of human resources, categorizing the area as lacking in physical accessibility and deeming it a priority block for service delivery improvement.

From this study, a database containing census information on 376 IHPs practicing in one of the ‘priority’ blocks of the Indian Sundarbans was made available to the study team. This census contained demographic details and information such as the location of the clinics, types of services provided, contact details and the average number of patients seen in a month. This database provided the sampling frame for our study. Using the principle of maximum variation, 35 IHPs were purposively sampled based on six characteristics—demographic characteristics (age, gender and education), service delivery (type of practice and average number of monthly patients) from two geographic locations (deltaic and non-deltaic) of the Indian Sundarbans (Appendix 2). We developed 23 categories from the above mentioned characteristics and selected 1–2 IHPs from each category. Of the 35 sampled IHPs, one migrated to another location during the fieldwork period and could not be interviewed, making the total sample 34 IHPs.

### Data collection

Data collection took place between January and April 2016. Intentional and unintentional bias may exist in the participants and researchers in all social research (Fusch *et al*., 2018). To minimize this, we implemented a Reflexive Diary (Lincoln and Guba, 1982) before and during the data collection. This involved keeping a note of the researchers’ perceptions and responses to the interview questions.

#### Ethnographic observation

Before the study began, two authors visited the Indian Sundarbans to carry out fieldwork for several months for ethnographic observation. The fieldwork for the current study took place after considerable familiarity with the study location had been gained. The first step in the study was a period of two months of general ethnographic observations (Kawulich, 2005). The observations were documented in the form of observational field notes during the visits to IHPs. The objective of this method was to gain insight into the social and physical context in which IHPs operate and to establish rapport with the villagers, IHPs and other members at the study site.

#### Qualitative ego-network method

**Figure 1.**
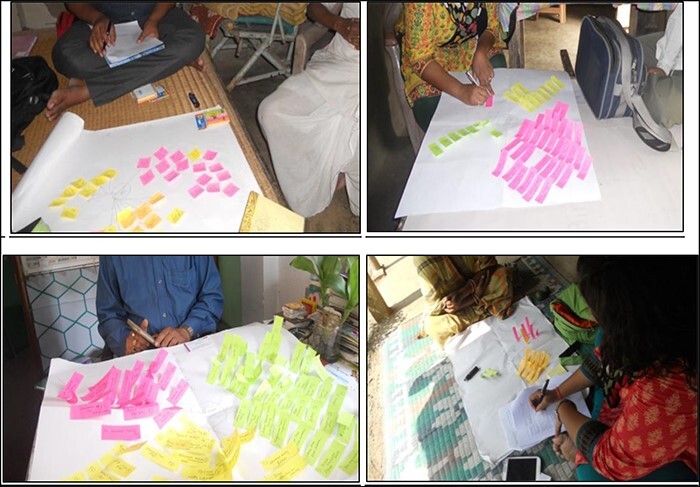
Participatory network mapping process

Note: faces of the participants are not shown to preserve anonymity

The study adopted the qualitative ego-network method (Wasserman and Faust, 1994; Emmel and Clark, 2009; Borgatti, 2011). It is a type of SNA that consists of a focal actor (‘Ego’) and its relationship with other actors (‘Alters’). The social network is drawn focusing on Ego and the list of actors the Ego is directly connected to. In our study, an IHP is the Ego and Alters are the other actors the IHP is directly connected to. The connections between the Ego and the Alter are called ‘Ties.’ We adopted a participatory approach to map the social network. A paper chart was used along with colourful sticky notes and pens to draw the social network of IHPs (Figure 1). We examined the social network of IHPs through three distinct enquiries: resource exchange, information and idea flow, and the nature of social support (Edwards, 2010). The analysis was based on types of relational ties by Wasserman and Faust (1994). The process began by explaining the study’s objectives, obtaining informed consent and recording the demographic details of the IHPs, including their name, age, gender and education, the mapping exercise then commenced. Following the mapping of ego networks, we assessed the strength of the ties with the Alters by measuring the frequency of support using a five-point Likert scale (ranging from 1-very frequent to 5-very rare) (Borgatti, 2011). For the purpose of analysis, we categorized the responses into ‘strong ties’ and ‘weak ties.’ Responses of ‘very frequent’ (1) and ‘frequent’ (2) were classified as ‘strong ties,’ indicating a high frequency of interaction. Conversely, responses of ‘rare’ (4) and ‘very rare’ (5) were classified as ‘weak ties,’ indicating a low frequency of interaction. The middle category, ‘somewhat’ (3), was excluded from the analysis to focus on the more definitive strong and weak tie relationships. This approach is consistent with methodologies employed in similar studies (e.g. Smith *et al*., 2018), where the exclusion of neutral or middle responses enhance the clarity and interpretability of the results. To comprehensively identify Alters, we adopted a life-history approach (Emmel and Clark, 2009), which involved discussing the IHPs’ journey into their profession and the individuals who supported and motivated them along the way. This approach revealed a broader range of Alters than a traditional name generator exercise would (Wasserman and Faust, 1994). During the mapping exercise, Alters were categorized as government, private or community actors. We also collected data on Alter’s attributes such as gender, age and years of work experience. To assess the duration and status of the ties, we asked whether the ties remained, if they did not, we enquired about their duration (in years). The qualitative ego-network method is particularly useful in exploring issues of intersectoral collaboration. This approach enables us to gain insight into the interconnected relationships and interactions among IHPs and other actors within a network, shedding light on the dynamics and complexities of collaboration across different sectors (Borgatti and Foster, 2003).

#### In-depth interview

In addition to the ego-network method, we conducted in-depth interviews (IDIs) to comprehensively explore various aspects of social networks (Emmel and Clark, 2009; Borgatti, 2011). This data collection approach focused on different types of relational ties (Wasserman and Faust, 1994) to provide a nuanced understanding of the provision of support, motives behind the relationship, the flow of resources, and the continuity of this support in both urgent and routine contexts. The qualitative component yielded rich narrative data, offering unique insights into the social networks of IHPs. On average, each mapping and interview session lasted approximately 1 hour and 35 minutes.

Data collection occurred in various settings, primarily in IHP clinics (21), with additional interviews taking place in their homes (12) and in the community (1). Only the researchers and participants were present during data collection. The research tools, including questions, prompts and guides, were initially developed in English, translated into Bengali and pilot-tested before finalization. The tools were tweaked based on pilot results, in particular, for the participatory mapping tool and the IDI guide where additional probing questions were added. All participatory mapping exercises and interviews were audio-recorded after obtaining written informed consent.

#### Data saturation

(Fusch and Ness, 2015; Glaser and Strauss, 2017) was focused on the level of analysis. The model of saturation was inductive thematic saturation, which is attained when no new codes or themes emerge from the data analysis (Saunders *et al*., 2018). The data saturation was examined using four criteria: (1) across different aspects of the study, (2) across study tools, (3) within categories of the study participants and (4) across the categories of study participants. Keeping these criteria in mind, a saturation grid was developed after the first data collection and coding round. The researchers performed a second round of data collection to further explore aspects that required more information.

### Ethical approval

The Institutional Committee for Ethics and Review of Research reviewed and approved the study protocol at the author’s institute in India (dated 27th June 2015). The study tools and methods designed for these purposes have taken into account the socio-cultural sensitivities and rights of the participants. All interview participants were informed of the objectives and procedures involved in the study. Written consent was obtained from all the participants regarding study participation. All participants read and were provided a copy of the consent form, which included the following information—(1) details of the study: objectives, methods and key areas of information sought, (2) voluntary participation: declaration of the right to refuse or accept participation, (3) confidentiality of information, and anonymity of the data, with only members of the research team having access to it (the data is to be used purely for academic and research purposes), (4) declaration of risks and benefits: no specific risk is posed by the study and no specific incentives would be provided for participation, (5) contact information: complete details of the interviewer, address, and e-mail and office phone number.

### Data analysis

We assigned unique identification numbers to each Ego and the Alters (network actors). Audio coding of interview data was done using Nvivo10 (QSR International Pty Ltd, version 10), incorporating deductive and inductive codes as per Bingham (2023). Deductive codes drew from social network literature (Wasserman and Faust, 1994). Reporting followed Consolidated Criteria for Reporting Qualitative Studies (COREQ) guidelines (Tong *et al.*, 2007). The coding process was carried out by two individual researchers. Appendix 1 contains the completed COREQ checklist. Kumu maps (Kumu Maps, 2024), an online visualization tool, was used for the social network visualizations.

## Results

### Characteristics of participants

The mean age of the participants was 48.5 years and there was only one female IHP. Most IHPs practiced allopathic medicine (94%), while a few specialized in homoeopathy (6%). Their average practice duration was 24.8 years. Twenty IHPs were the sole practitioners in their families, influenced by relatives such as their fathers or uncles. The majority, i.e. 25 (53%) had received training or certificates from IHP associations and NGOs in areas including drugs, pharmacy, community health and alternative medicines (Table 1). They developed expertise through experience and participation in conferences/training in fields such as child health, women’s health, dentistry, ayurveda, orthopaedics and surgery. Typically, IHPs worked from rented spaces, with only three owning clinics, some of which were supported by CBOs.

**Table 1.** Characteristics of participants

Particulars	*n* = 34
Age (years)	
Mean	48.5
≤30	6%
31–50	47%
51–60	29%
≥60	18%
Highest level of education	
Primary (5–10 years)	6%
Secondary (11–15 years)	71%
Higher Secondary (16–17 years)	18%
Graduate (17–20 years)	6%
System of medicine practiced	
Allopathy	94%
Homoeopathy	6%
Years of practice	
Mean	24.8
≤ 10 years	9%
11–30 years	59%
31–40 years	18%
> 40 years	15%
Certificate course/diploma	
Yes	53%
No	47%
Mean number of villages covered	6.5

### Social network of IHPs

Through the mapping exercise and life-history approach we found that the 34 IHPs had a total of 1362 ties with diverse health system actors (Alters). Such actors were grouped into three broad categories which included: 814 private sector actors (60%), 339 community actors (25%) and 209 government health actors (15%). The network with all three categories of actors is elaborated in the subsequent sections.

#### Ties with private sector actors

**Figure 2.**
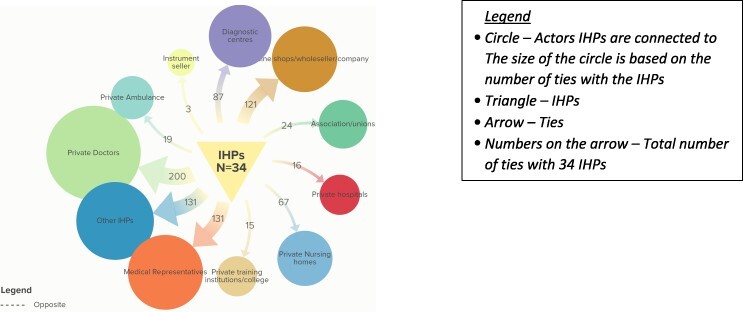
Total number of ties informal healthcare providers (IHPs) have with various private sector actors

The social network of IHPs with private sector actors is very dense with multiple actors connected with one another. They constitute the largest proportion, at 814 (60%), of the actors connected with IHPs. The majority of ties are with private doctors (200; 25%), other IHPs in the region (131; 16%), medical representatives from pharmaceutical companies (131; 16%), medicine shops (121; 15%) and diagnostic centres (87; 11%). The remaining ties include those with private nursing homes, IHP associations, ambulance drivers (private), private hospitals, training institutions, and suppliers of drugs and medical instruments (Figure 2). Almost all IHPs have ties with medicine shops, pharmaceutical companies, diagnostic centres and private doctors (Table 3). IHPs said that diagnostic centres and nursing homes incentivize IHPs to refer patients to them; however, the IHPs do not request additional money from the patient’s family to refer and accompany them to such destinations.

IHP 3, male said *‘we don’t charge extra money from the patient family, as it may increase the cost for the them’*.

##### Type of support with private sector actors

The relational ties (Wasserman and Faust, 1994) with private sector actors involve seeking advice on the line of treatment and referral, transfer of material resources, capacity building, movement between places and behavioural interactions. Many IHPs said that representatives from pharmaceutical companies visit them and keep them updated on the latest drugs, and supply medicines to them. Pharmaceutical companies staged the majority of training to the IHPs and play a significant role in providing them with updates on the latest drugs. During the fieldwork phase, we have also observed medical representatives waiting outside the IHP clinic as they do for formally qualified doctors.

Local pharmacists share knowledge regarding drugs, take orders over the phone and deliver to the IHP’s doorstep. IHPs often refer patients with ‘critical’ symptoms to hospitals. Referrals are especially prominent among IHPs who practice allopathic medicine. An IHP (IHP 21, male) puts it, ‘*it is a way to develop a good relationship with the doctor in order to seek advice in the future’*. IHPs interact with doctors for patient care during referrals, often accompanying patients during emergencies, fostering trust. The choice of referral destination, public or private, depends on the financial situation of the patient. IHPs refer patients to private hospitals in Kolkata (the nearest metropolitan city) due to their connections. For patients covered by the state-funded insurance scheme, IHPs refer and accompany them to the approved facilities. IHPs have established robust referral processes, ensuring that, with their support, patients can access specialized doctors in nearby towns, public hospitals, medical colleges and private hospitals in the cities when needed.

Diagnostic centres collaborate with IHPs, designating their clinics as sample collection centres, and deputing their agents to collect samples. Some well-known diagnostic centres in eastern India have been mentioned by the IHPs as being a part of their network. IHPs may receive financial compensation, typically a percentage of the test cost for referred patients. Appointments are often scheduled via phone.

*Diagnostic centres in nearby towns send their agents to visit us and collect the samples for tests, which help us in preliminary diagnosis of the patient’s conditions being located in such a remote region* (IHP5, male).

Private nursing homes run by qualified healthcare providers are gradually increasing in the Indian Sundarbans. These are the newer Alters in the social networks of the IHPs. They seek clinical knowledge and skills from such Alters, and in turn, they may refer patients to private nursing homes.

The local associations of the IHPs provide a forum for the coming together of the IHPs to advocate for their rights and gain recognition. Being a member of the association accords them dignity, the associations also act as defence in the event of IHPs being subject to harassment by the authorities or local people.

#### Ties with community actors

**Figure 3.**
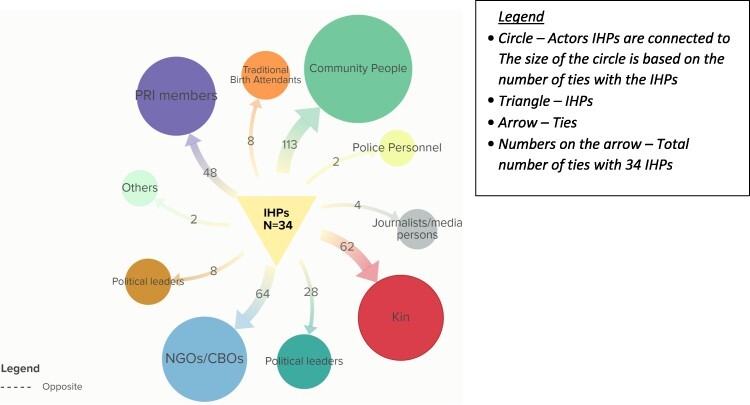
Total number of ties informal healthcare providers (IHPs) have with various community actors

Note: PRI, Panchayati Raj Institution (Local self government in India); NGO, Non-Governmental Organisation; CBO, Community Based Organisation

Community actors constitute the second largest category of ties in the social network of the IHPs, comprising 339 (25%) of the total ties. The majority of ties are with community people at 113 (34%), followed by CBOs at 64 (19%) and Kin at 62 (19%) (Figure 3). The majority of the IHPs possess ties with community people (82%), Kin (79%) and with Panchayati Raj Institutional members (PRIs)—the local administrative units (76%) (Table 3).

##### Type of support with community actors

The type of relational ties (Wasserman and Faust, 1994) with community actors are biological relationship or kinship (Kin of IHPs), transfer of material and resources, behavioural interaction, movement between places, formal relations and association or affiliation. The ties are very strong (Table 2). Most ties involve local community members assisting IHPs in daily tasks and facilitating patient care. CBOs offer training, health camp participation and infrastructure support, strengthening the integration of IHPs in the local community. Kinship ties involve family members providing motivation, financial aid and constant support, fostering trust and reliability due to their unwavering availability, flexibility and emotional support in emergencies.

**Table 2.** Status and strength of ties

	Status of social ties		Strength of the social ties	
Type of actors	% Active ties	% Inactive ties	Strong ties	Weak ties
Private sector actors	**81%**	**19%**	**62%**	**21%**
Private doctors	77%	23%	60%	24%
Other IHPs	89%	11%	64%	14%
Medical representatives	78%	22%	53%	31%
Medicine shops/wholesaler/company	88%	12%	73%	17%
Diagnostic centre	89%	11%	63%	16%
Private nursing home	87%	13%	63%	19%
IHP association/unions	63%	38%	75%	17%
Private ambulance drivers	89%	11%	74%	16%
Private hospital	69%	31%	31%	56%
Private training institutions/college	7%	93%	73%	27%
Instrument seller	67%	33%	0	67%
Community actors	**83%**	**17%**	**74%**	**11%**
Community people	93%	7%	66%	18%
NGO/CBO	70%	30%	63%	16%
Kins	73%	27%	92%	2%
PRI	94%	6%	77%	6%
School teacher	86%	14%	73%	12%
Others	100%	0%	0%	0%
Police	100%	0%	100%	0%
Political leader	75%	25%	88%	13%
Journalist/media	50%	50%	75%	0%
Traditional Birth Attendants	88%	13%	38%	0%
Government health actors	**78%**	**22%**	**70%**	**15%**
Government doctors	77%	23%	75%	9%
Frontline health workers	91%	9%	71%	23%
Government health centre/hospital	79%	21%	61%	20%
Government ambulance	67%	33%	67%	0%
Hospital staff	33%	67%	67%	33%
Institutes/association	33%	67%	33%	33%
Total	**81%**	**19%**	**66%**	**18%**

Note(s): the status of social ties was at the time of data collection i.e. 2016.

**Table 3.** Percentage of IHPs have at least one tie with the given actors and average duration of ties

Type of actors	% of IHPs with ties (*n* = 34)	Average duration of ties (in years)
Private sector actors		
Medicine shops/wholesaler/company	100%	13.91
Diagnostic centre	100%	10.66
Private doctors	97%	10.01
Other IHPs	91%	19.67
Medical representatives	79%	7.56
Private nursing home	71%	9.72
IHP association/unions	53%	12.67
Private ambulance drivers	41%	5.79
Private training institutions/college	35%	5.00
Private hospital	26%	11.81
Instrument seller	9%	18.00
Community actors		
Community people	82%	22.66
Kins	79%	34.85
PRI	76%	25.09
NGO/CBO	65%	9.66
School teacher	44%	33.25
Political leader	18%	27.88
Traditional birth attendants	15%	17.63
Others	6%	20.50
Police	3%	15.00
Journalist/media	9%	10.50
Government health actors		
Government doctors	100%	10.27
Government health centre/hospital	79%	15.89
Frontline health workers	50%	12.43
Institutes/association	9%	8.67
Government ambulance	6%	2.00
Hospital staff	6%	21.00
Average		**14.71**

During the fieldwork, we observed that reaching a nearby health facility takes 4–5 hours from the village due to transportation challenges, including crossing the river(s) and motor van rides. Pregnant women rarely undertake this journey. IHPs are the preferred choice, offering first aid and services and utilizing connections with government and private providers.

An IHP shared, *‘In the last few years, I have delivered 26 babies on a boat. When patient comes to them at the last hour or while crossing the river, or when the boat gets dysfunctional or runs out of fuel. Otherwise, the women would have died on the way due to heavy bleeding*’ (IHP 16, male).

Due to strong community ties and dedication to this profession, payment flexibility is offered, and monetary gains are not always sought from the patient’s family. IHP 8 male said, *‘In this profession, we find satisfaction and joy in witnessing the smiles on our patients’ faces, regardless of financial compensation.’*

Through ethnographic observation, we have observed that IHPs are the first point of contact as formal healthcare facilities are not close by. A woman in the community (age 46) remarked that *‘We always visit the “doctor” (IHP) for any health-related issues because he is always available and listens to us. He is very well-behaved. Once my sister-in-law was ill and we did not know where to take her. That time we contacted this doctor, and he took us to a nearby city and helped us in consulting a doctor there. He is a saviour’*.

IHPs collaborate with local political leaders who facilitate patient referrals to nearby government hospitals, offer financial backing for health camps and attract attendees. An IHP described how they are asked by the local administration to convince villagers to attend the polio camp:

*Whenever polio camps/campaigns were organized in the village, the Panchayat Pradhan (elected head of the local body) always asked us to convince villagers to visit the camps, because they respect us* (IHP 3, male).

#### Ties with government health actors

**Figure 4.**
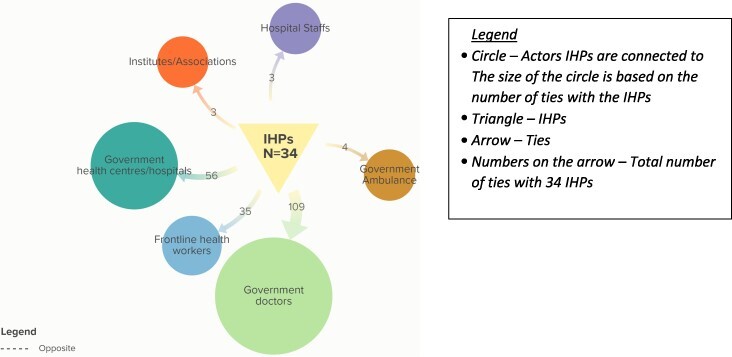
Total number of ties informal healthcare providers (IHPs) have with various government health actors

This is the third category of actors in the IHP network. Government doctors (52%), hospitals/health centres (27%) and frontline health workers (17%) are major actors in the network, connected to several IHPs with strong ties (Figure 4).

Types of support with government health actors

IHPs maintain various relational ties (Wasserman and Faust, 1994) with government health actors, which can be categorized as formal relations, kinship and advice, and referral networks. All IHPs have links with government doctors (Table 3). The majority started maintaining formal relations with government actors by referring patients/taking them to those qualified doctors and hospitals for treatment when the situation became critical, also seeking advice from qualified doctors over the phone and providing vital assistance to communities during emergencies. These relationships are underpinned by several factors, e.g. rapport developed over the years while taking patients for check-ups.

*I always refer patient to “government hospital” for critical cases, I also accompany them to the hospital and do administrative duties because patient’s family relies on us, which also helps us in developing good relations with the doctors* (IHP8, male).

An illustrative example highlights the critical role of these connections.

*One day I received a call from a family where they were conducting delivery of a baby with the help of a traditional birth attendant. The situation was very critical, and they asked me to visit. When I visited, I observed that it is a breech position. I suggested they take the woman to some nursing home or hospital immediately, but due to low tide in the river, and unavailability of boat services they were unable to do so. Hence, I took the risk because I had experience with one or two breech deliveries. I called my senior gynaecologist in the city. I explained to him the situation and he guided me. I took the baby out then and saw there was another baby inside. Even the family did not know about the second baby as sonography had not been done. The doctor said, listen to me carefully and do it. I did accordingly and saved the mother’s life* (IHP14, male).

Numerous government doctors with various specializations, including orthopaedics, gynaecology and paediatrics, serve as mentors for IHPs. On asking how these relations were developed, IHPs mentioned over the years they have been taking patients (villagers) for treatment to nearby hospitals (for critical cases) and thereby they developed relationships with the qualified doctors.

*The hospital does not provide bed to the patient, if I don’t go with them, also if we go with the patient family, we can help them in paperwork at hospital* (IHP18, male).

Some doctors practice privately in their village once a week/month. Additionally, meeting them during training organized by pharmaceutical companies helps build connections. Some of them are also relatives of IHPs. These IHPs acquire valuable skills by working alongside these qualified doctors, learning procedures such as administering intravenous fluids, injections, catheter placement and conducting deliveries. Recognizing medical emergencies is also a crucial skill learned from these experienced doctors. IHPs actively seek out these specialists for guidance. Strong, family-like relationships often form with these qualified doctors, especially those who practice locally. As an IHP said of a doctor with postgraduate qualifications, ‘*He holds a MD degree. He is an expert with better knowledge of disease and medicine. Therefore, I prefer him’* (IHP8, male). Such collaborative ties with specialists and healthcare professionals contribute significantly to the provision of healthcare services in the region.

### Strength of the ties

Usually, there are two types of ties (strong and weak) in SNA. The study revealed a significant predominance of strong ties within the network, constituting 66% of all connections. In our study, 895 strong ties were reported, comprising most of the connections with Alters (66%). Approximately 244 (18%) of the ties were categorized as weak (Table 2).

#### Strongest ties

The strongest ties in the private sector are with IHP associations (75%), private ambulance drivers (74%), medicine shops/wholesalers/companies (73%) and private training institutions (73%). Among the community actors, the strong ties are with the police (100%), kins (92%) and political leaders (88%). Among government health actors, the strongest ties are found with government doctors (75%) and frontline health workers (71%) (Table 2).

#### Weak ties

The weakest ties in the private sector actors are with instrument sellers (67%), private hospitals (56%), private training institutions (27%) and private doctors (24%). The reason stated by IHPs is mainly the nature of the interactions being more transactional or occasional rather than based on frequent collaboration or personal trust. Among the community actors, the weak ties are with community people (18%) and NGOs/CBOs (16%). Among the government health actors, 15% of ties are found to be weak with hospital staff (33%), and institutes and associations (33%) (Table 2).

### Duration of social ties

On average, IHPs have more than a decade of relationships with the health system actors across the categories (14.7 years). They demonstrate long-standing relationships with community actors, with an average spanning more than two decades (23 years), while the ties with private and government actors are comparatively shorter, with an average duration of 12 years each. The duration of ties varies across the sub-categories (Table 3).

### Changes in the social network over time

IHPs possess dynamic ties with health system actors, which change over the years with the exit of some actors and entry of new ones. Most of the ties are active, at 1106 (81%), across the three types of Alters, 273 (83%) with community actors, 668 (81%) with private and 165 (78%) with government health system actors. Actors may exit and enter the network over time. Due to changing practices in seeking healthcare in the region, some ties are gradually becoming either weak or ceasing to exist altogether, e.g. the ties between traditional birth attendants have ended due to the introduction of government programmes to promote institutional delivery. Only a few IHPs (15%) have ties with Traditional Birth Attendants (TBA)s at present, which is 1% of the total ties (1362) (Table 3). Due to religious reasons they have to maintain those relationships. An IHP (IHP2, male) shared, *‘In some communities the household heads do not allow us to touch the pregnant woman due to religious reason, they just allow us to measure blood pressure, give injections and saline. I cannot give injections if I do not know the status of the woman by physically examining her. In such cases TBAs support us by checking and informing. Based on that, we provide injection to induce labour. That is how we have to work in these areas and after the introduction of JSY (Jannani Suraksha Yojana, government scheme), we send all pregnant women to the hospital and nursing home, we don’t do anything’*.

On the other hand, new actors may enter the network, e.g. Accredited Social Health Activist (ASHA)s were introduced as frontline health workers for the maternal and newborn health programme by the government of India through the National Rural Health Mission in India in 2005 (Government of India, 2005). They motivate women to seek care during pregnancy and opt for institutional deliveries. ASHAs are the new actors in the social network of IHPs; however, there is tension in the ties between the two. As an IHP said, *‘We always support their work, but they don’t support us. Nor do they come to us’* (IHP 12, male). The promotion of institutional delivery by ASHA impacted the traditional practices of delivery at the community and by the IHPs. This was a big shift as IHPs rarely conduct home deliveries after the ASHAs have been introduced. Instead, they prefer to provide antenatal care services to pregnant women and refer them to designated healthcare facilities for childbirth. An IHP stated:

*‘Nowadays we have been instructed not to perform any delivery, so I do not take up pregnancy cases anymore. I only conduct the pregnancy test. If the woman tests positive (for pregnancy), then I just inform the ASHA or refer the pregnant woman to a hospital’* (IHP28, male). IHPs perceive themselves as more knowledgeable, experienced and possessing greater reputation in the community than the ASHAs. An IHP shared, *‘We have been addressing the community health issues for a long time, even before there were no hospitals and health workers around, we have so much of experience but, still government does not consider us as health workers, rather they introduced ASHAs who are new, younger, and have less experience. There was a time when these girls used to come to us and now, they don’t support us’* (IHP 24, male).

## Discussion

### IHPs in practice: informal yet interconnected

Our research builds upon a well-established body of literature by further investigating the social network of IHPs through a qualitative SNA, thereby distinguishing our research from existing studies on IHPs. Our study findings highlight the intricate social networks of IHPs and help understand the complex relationship between IHPs and qualified medical practitioners, as emphasized by Sudhinaraset *et al*. (2013). Nair *et al.*, (2019b) highlight the potential for future research and intervention in exploring the nexus between allopathic doctors and pharmaceutical company representatives. Our study affirms the observations of other scholars (George and Iyer, 2013; Gautham *et al*., 2021), despite operating illegally and informally, the IHDs ‘expertise, service networks, reputations and success are deeply embedded in the formal health systems and communities to which they belong’. Our study contributes to the existing body of literature by examining their social networks in greater depth, building upon insights from previous studies such as those by Chandra and Bhattacharya (2019), Nair *et al.* (2019a, b) and Gautham *et al*. (2021). While previous research has provided valuable insights into the economic and social dependencies of IHPs with other actors, our study adds new dimensions to this understanding by elucidating the intricacies of their relationship with various actors in the health systems. Previous studies have highlighted the ties of IHPs with mainly private and community actors; our study reveals their relationship with government health actors as well.

From our study, we establish that IHPs do not operate in isolation; they maintain strong and active linkages with government, private sector and local communities (Datta, 2013; Gautham *et al*., 2021). These networks signify the support for IHP that allows them to operate in a supportive ecosystem, which they have sustained for their professional growth while addressing community health needs (George and Iyer, 2013). This is consistent with the findings from another study in India (Gautham *et al*., 2014), which found doctors and medical representatives of pharmaceutical companies were sources of knowledge for IHPs.

Our study revealed findings regarding the strength of IHPs ties with health system actors. Most of the ties are strong in nature (66%) and 18% ties are weak. There are several merits of these strong ties such as: Enhanced Collaboration and Support, i.e. strong ties with IHP associations and private training institutions indicate a robust network for continuous professional development and mutual support among IHPs, which can lead to improved skills and knowledge, ultimately enhancing the quality of care provided. Effective Resource Mobilization, i.e. strong ties with medicine shops/wholesalers and private ambulance drivers facilitate quick and efficient access to medical supplies and emergency transport services, which is vital for timely patient care. Community Trust and Engagement, i.e. strong ties with community actors such as police, kins, and political leaders reflect high levels of trust and engagement with the community, which can enhance public health initiatives, community health education and support during health crises. This finding is supported by research in Nigeria, where IHPs have been recognized for their essential role in community health, often filling gaps left by formal health services. Strong community ties are vital for gaining trust and ensuring effective healthcare delivery in underserved areas (Onwujekwe *et al*., 2022). Integration with Formal Health Sector, i.e. strong ties with government doctors and frontline health workers suggest a good level of integration between IHPs and the formal health sector, which can improve referral systems, collaborative patient care and the overall improvement of health service delivery. These findings are mirrored in Kenya where the initiatives to train IHPs in mental healthcare and integrate them into the formal referral system have strengthened these relationships, demonstrating the importance of collaborative ties for enhancing healthcare delivery at the community level (Musyimi *et al*., 2017).

While strong ties are instrumental in fostering collaboration and mutual support, it’s also important to recognize the value of weak ties, as noted by Granoveter (1973) and Donev (2005) and Granoveter (1973). The dominance of strong ties with government health actors and community actors suggests robust, supportive networks that ensure reliable service delivery and community trust. However, the fewer weak ties may limit the introduction of novel information and practices from outside the immediate network. There are several implications of weak ties such as Transactional Relationships, i.e. weak ties with instrument sellers and private hospitals suggest that interactions are primarily transactional, which can hinder the development of trust and long-term collaboration. This may affect the continuity of care and the willingness to share critical health information. Fragmented Health Services, i.e. weak ties with hospital staff and health institutes suggest a fragmentation in the health services network, which can lead to poor co-ordination, inefficiencies in patient referrals and gaps in comprehensive care. Direct patient interactions, i.e. the weak ties of IHPs with CBOs is consistent with findings from Bangladesh, which shows informal providers are less engaged with broader community organizations and more focused on direct patient interactions. This limited engagement can restrict the flow of new information and innovations from these sectors to the IHPs (Sujon *et al*., 2023).

Understanding these dynamics provides valuable insight for optimizing resource utilization and enhancing collaboration within the network. Strategies may include leveraging strong ties for deeper engagement and resource mobilization, while also nurturing weak ties to broaden access to diverse resources and expertise.

The resilience of IHPs is notable, as evidenced by their ability to adapt and evolve over time. Our findings demonstrate the average duration of the IHP social ties is more than decade. Hence, their social ties serve as a backbone, demonstrating their ability to endure and prosper, which is consistent with the findings of George and Iyer (2013), Sudhinaraset *et al*. (2013), Chandra and Bhattacharya (2019) and Gautham *et al*. (2021). These enduring ties are critical in regions with limited access to formal healthcare facilities, as IHPs serve as the first point of contact during emergencies, offering flexible payment options and garnering strong community trust (Naicker *et al*., 2010; Sharma, 2015; Rao and Rao, 2017; Drennan and Ross, 2019; Tweheyo *et al*., 2019; Karan *et al*., 2021; Nair *et al*., 2022). This phenomenon has been observed not only in our study but also in various contexts across Asia and Africa (Bhuiya, 2009; Mahmood *et al*., 2010; Sudhinaraset *et al*., 2013; Gautham *et al*., 2014; Onwujekwe *et al*., 2022).

### The role of IHPs social network in intersectoral collaboration

Intersectoral collaboration emerges as a strategic approach to address the challenges faced by IHPs, and the health systems such as training, regulation and quality control (Sudhinaraset *et al*., 2013; Gautham *et al*., 2014; 2021; Kumah, 2022). However, there is a lack of understanding regarding the interactions between actors that lead to intersectoral collaboration (Chiari *et al.*, 2023), and our research sheds light on the potential social ties of IHPs as strategic assets in advancing the UHC agenda in low- and middle income countries (LMIC) (Kumah, 2022). The recent report of the WHO (2023) states the importance of multisector collaboration and partnership to face the challenges in health system delivery post-pandemic. The majority of studies suggest building capacity will address the quality issue (Sun *et al.*, 2022), while very few measured the retention of knowledge after training (Shah *et al.*, 2011). In our study we have seen that the IHPs acquire continuous knowledge through their social ties with government and private doctors and pharmaceutical companies, which helps them in practice. Hence, strengthening the existing ties through collaborative efforts between public and private actors healthcare authorities (WHO, 2020), educational institutions and CBOs can strengthen knowledge and skills while ensuring adherence to healthcare standards (WHO, 2018). The resources embedded in the social relations among people and organizations can facilitate co-operation and collaboration (Donev, 2005; Chiari *et al.*, 2023). This is consistent with the study by Borgatti and Foster (2003), which highlights the utility of ego-network methods in uncovering the structural and relational aspects of intersectoral collaboration, providing valuable insight for both researchers and practitioners. These extensive affiliations hold promise for enhancing community-based interventions, strengthening the existing referral mechanism and sharing of resources aimed at achieving UHC (George and Iyer, 2013; Nayak *et al*., 2022). Enhancing the quality of IHPs can significantly contribute to extending UHC in LMICs (Kumah, 2022; Coveney *et al*., 2023). However, challenges such as regulatory barriers, lack of standardized training and co-ordination issues pose significant obstacles to seamless intersectoral collaboration (J. van Rensburg and Brooke-Sumner, 2023; Nutbeam, 1994). Furthermore, we recognize the impact of national policies and programmes on changing social networks within the health system. Coordination and intersectoral collaboration are essential to address conflicting roles with government actors and ensure comprehensive healthcare access.

### Policy ambiguity and pragmatic solutions

In India and other South Asian countries, the policy landscape regarding IHPs is complex, reflecting the moral dilemma of unqualified providers serving underserved populations. Pragmatic solutions are imperative, particularly in areas facing continued workforce shortages. The COVID-19 pandemic has further highlighted the importance of IHPs in areas where formal health systems struggle with workforce shortages and absenteeism (Tweheyo *et al*., 2019; WHO, 2020). We suggest systems and policies must acknowledge the existence of considerable human resources such as IHPs (Bloom *et al*., 2011) on the ground by leveraging their strong social ties with various health system actors to improve health service delivery to prepare for future pandemics (Bhuiya, 2009; Kanjilal *et al*., 2010; Sudhinaraset *et al*., 2013; Islam *et al*., 2014; Sieverding *et al*., 2015; Nayak *et al*., 2022; WHO, 2020). Achieving UHC in underserved areas may require a re-evaluation of the role of IHPs and integration into the national health programmes to formalize collaboration with other potential actors such as formal frontline healthcare workers, school teachers, local administration, influential community people and private actors like pharmaceutical companies.

Sustained intersectoral collaboration can maximize the strategic potential of IHPs’ social networks, offering a path towards realizing UHC in regions that have long struggled with healthcare access (Kumah, 2022). The implications of our findings in relation to existing policy ambiguities surrounding IHPs are significant. Despite their integral role in healthcare delivery, policy inaction persists, reflecting the complex moral and regulatory dilemmas associated with their practice. Conflicts of interest between formal and informal healthcare sectors further exacerbate these challenges, hindering efforts to formalize collaboration and regulate the practice of IHPs (Rahman-Shepherd *et al.*, 2021) The recommendations made in our study, while not novel, underscore the urgent need for pragmatic approaches to address these longstanding issues. The social networks of IHPs, highlighted in our study, present evidence to rethink ways in which health services can be delivered in disadvantageous regions. Our study findings can hold true for other contexts of underserved hard-to-reach rural areas where the communities rely on IHPs for healthcare access. IHPs are present in urban areas, but not as robustly. As urban and rural contexts are different, the type of actors can be varied, hence the transferability of our findings to the urban context is not suggested. The absence of the perspectives of the Alters is a limitation of our study. The ties were self-reported by IHPs as we did not collect data from the Alters to validate those ties of the IHPs due to time and resource constraints. Future research can delve further into the understanding from the perspective of the Alters of the reason(s) for sustained relationship with IHPs and how they perceive the opportunity of collaboration with IHPs to play a role in achieving UHC.

## Conclusion

Leaving no one behind is the promise of Sustainable Development Goals 2030. The pursuit of UHC demands a meticulous examination of healthcare delivery systems, encompassing formal and informal sectors alike. Our study underscores the indispensable role of IHPs and their social networks in advancing equitable healthcare access. Our findings are grounded in empirical evidence and align with established literature on IHPs’ contributions to healthcare delivery. The WHO recognizes both formal and informal health entities, as evidenced in the public-private mix (PPM) for TB prevention, underscoring the relevance of our study within the broader global health discourse. The rootedness of the IHPs in the local communities while being connected to diverse government, private and community healthcare actors makes them potential fulcrums in the sustainability of community health through intersectoral collaboration. The evidence from this and other studies acknowledges the presence of IHPs in the health systems, highlighting both their dedication and service to the communities as well as challenges associated with their practice. This body of research provides insights that can inform policymakers on how to integrate IHPs into formal health systems, ensuring that they adhere to healthcare standards and regulations through strategic intersectoral collaboration in LMICs.

Though these recommendations are not novel, as they have been offered in previous studies, emphasizing the need for strategic integration of IHPs into formal health systems, our study contributes new insights to existing knowledge by examining the role of social networks in facilitating intersectoral collaboration, delving into the intricate relationships and connections among IHPs and various actors within the health systems. Through our analysis, we identify opportunities for intersectoral collaboration to address regulatory issues, improve training standards, and enhance adherence to healthcare protocols among IHPs. By fostering collaboration between healthcare authorities, educational institutions and CBOs, policymakers can develop comprehensive strategies to integrate IHPs into formal health systems effectively. Leveraging strong ties with government, community and private sector actors can facilitate resource exchange, knowledge sharing and collaborative efforts aimed at enhancing healthcare delivery and achieving UHC objectives.

## Figures and Tables

**Figure:**
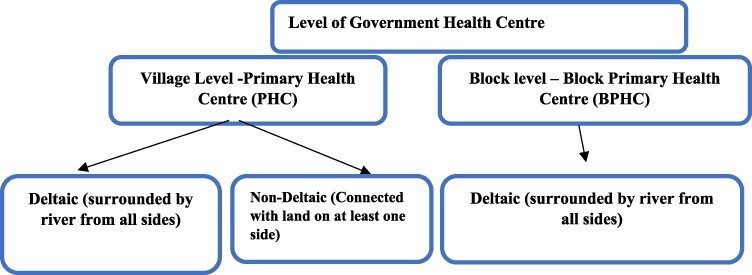
Purposive sampling based on the Maximum Variation Principle

IHPs were selected from each of the above types followed by seven criteria–demographic characteristics (age, gender and education) and service delivery (type of practice and average monthly patients).

## Data Availability

The data underlying this article can be shared upon reasonable request to the corresponding author.

## Abbreviations

ASHA = Accredited Social Health Activist

AHPSR = Alliance for Health Policy and Systems Research

BPHC = Block Primary Health Centre

CBO = community-based organization

COREQ = Consolidated Criteria for Reporting Qualitative Studies

GIS = Geographic Information System

HSG = Health System Global

IDI = In-Depth Interview

IHP = Informal Health Provider

LMIC = Low and Middle Income Countries

NGO = Non-Governmental Organisation

PRI = Panchayati Raj Institution

SNA = Social Network Analysis

TB = Tuberculosis

TBA = Traditional Birth Attendant

UHC = Universal Health Coverage

WHO = World Health Organization

## Reflexivity statement

All four authors are from middle-income countries, with three based in India and one in Georgia. We have wide-ranging international experience of health systems (and other pertinent issues). We are aware that universal health coverage can be particularly challenging in poor and underserved areas where formal healthcare delivery is severely compromised. One of us (S.M.) has grown up in the rural village and has first-hand experience of the hardships in accessing healthcare. The others have more privileged upbringing. R.B. and M.M. hail from professional middle-class families based in urban India. G.G. is from Georgia, President of Curatio International Foundation and Associate Professor at Ilia State University and has worked on health policy and systems issues globally for past three decades. He was invited to mentor R.B. and support her under the Health System Global’s initiative ‘Publication Mentorship Program for First-Time Women Authors in the Field of HPSR’. The research question was primarily a matter of academic curiosity for us. Our protracted engagement with the study site has made us sensitive to the issue as well.

Prior to the study, we were aware about the considerable presence of the IHPs in the Indian Sundarbans. We viewed them as local people with little or no qualifications to provide the services they do. We believed that IHPs provide ad hoc services in the area, until more professional help comes by (if at all). We also believed that IHPs operated on their own, using the knowledge and skills that they had inadvertently picked up from qualified healthcare providers. This study has debunked much of what we thought about the IHPs in the Indian Sundarbans.

## Appendix

**Appendix 1.** Completed Consolidated Criteria for Reporting Qualitative Studies (COREQ) checklist

No.	Item	Guide
**Domain 1: research team and reflexivity**		
**Personal characteristics**		
	Interviewer/facilitator	Which author/s conducted the interview or focus group*?*
		First author (R.B.) and fourth author (S.M.)
	Credentials	What were the researcher’s credentials? e.g. PhD, MD
		First author (R.B.)— PGDRM (equivalent to MBA), MSW
		Second author (M.M.)—Master’s in Arts (MA), Doctor of Philosophy (PhD)
		Third author (G.G.) —Doctor of Medicine (MD), Doctor of Philosophy (PhD)
		Fourth author (S.M.) —Masters of Social Work (MSW)
	Occupation	What was their occupation at the time of the study?
		First author (R.B.)—Research Officer at IIHMR University, India
		Second author (M.M.)—Assistant Professor at IIHMR University, India
		Third author (G.G.)—Associate Professor at Ilia State University, Georgia
		Fourth author (S.M.)—Research Assistant at IIHMR University, India
	Gender	Was the researcher male or female?
		First author (R.B.)—Female
		Second author (M.M.)—Female
		Third author (G.G.)—Male
		Fourth author (S.M.)—Male
	Experience and training	What experience or training did the researcher have?
		First author (R.B.)—She is a Monitoring and Evaluation professional with more than eight years of experience in research, design, and implementation of result-based M and E systems skilled in innovative cutting-edge participatory methodology and qualitative research
		Second author (M.M.)—She is a Professor in Public Health. She has a background in Sociology. Qualitative research is a major area of her teaching and research
		Third author (G.G.)—He is a professor and doctor who brings more than 25 years of experience, thorough knowledge and expertise in health systems, policy and financing issues. He has worked in over two dozen countries in Eastern Europe, Central Asia, Africa, the Caribbean and the Middle East
		Fourth author (S.M.)—He is a social work professional with eight years of experience in programme implementation, qualitative research, data collection and analysis
**Relationship with participants**		
6.	The relationship established	Was a relationship established prior to study commencement?
		Yes, two authors had visited the communities prior to the study for various professional reasons. This time was spent to build rapport. They also carried out ethnographic observations prior to the study
7.	Participant knowledge of the interviewer	What did the participants know about the researcher, e.g. personal goals and reasons for doing the research?
		Yes, the objectives of the research were explained to the study participants during the rapport-building phase. These were subsequently reiterated during the process of informed consent taking
8.	Interviewer characteristics	What characteristics were reported about the interviewer/facilitator, e.g. bias, assumptions, reasons and interests in the research topic?
		The reason for conducting the study was shared with all the participants. Researchers’ current role was also shared
**Domain 2: study design theoretical framework**		
9.	Methodological orientation and theory	What methodological orientation was stated to underpin the study, e.g. grounded theory, discourse analysis, ethnography, phenomenology, content analysis?
		Ethnography
**Participant selection**		
10.	Sampling	How were participants selected, e.g. purposive, convenience, consecutive, snowball?
		Purposive sampling using the maximum variation principle
11.	Method of approach	How were participants approached, e.g. face-to-face, telephone, mail, e-mail?
		Face-to-face
12.	Sample size	How many participants were in the study?
		34 participants
13.	Non-participation	How many people refused to participate or dropped out? Reasons?
		Total sample was 35. One sampled IHP had migrated to another place at the time of fieldwork and could not be interviewed
**Setting**		
14.	Setting of data collection	Where was the data collected, e.g. home, clinic, workplace?
		Data was collected in a place selected by the participants which includes home as well as clinic
15.	Presence of non-participants	Was anyone else present besides the participants and researchers?
		No
16.	Description of sample	What are the important characteristics of the sample, e.g. demographic data, date?
		Demographic characteristics about the participants such as age, gender, education. More details about the sampling are contained in Appendix 2
**Data collection**		
17.	Interview guide	Were questions, prompts and guides provided by the authors? Was it pilot tested?
		Yes, pilot testing of the tools was conducted prior to the data collection in two different locations
18.	Repeat interviews	Were repeat interviews carried out? If yes, how many?
		Some interviews were repeated for data saturation
19.	Audio/visual recording	Did the research use audio or visual recording to collect the data?
		Data was audio recorded
20.	Field notes	Were field notes made during and/or after the interview or focus group?
		During the visits
21.	Duration	What was the duration of the interviews or focus groups?
		Variable, an average of 1 hr 35 min and 29 sec
22.	Data saturation	Was data saturation discussed?
		Yes
23.	Transcripts returned	Were transcripts returned to participants for comment and/or correction?
		No
**Domain 3: analysis and findings data analysis**		
24.	Number of data coders	How many data coders coded the data?
		Two
25.	Description of the coding tree	Did authors provide a description of the coding tree?
		Yes
26.	Derivation of themes	Were themes identified in advance or derived from the data?
		Both
27.	Software	What software, if applicable, was used to manage the data?
		NVivo 10
28.	Participant checking	Did participants provide feedback on the findings?
		No
Reporting		
29.	Quotations presented	Were participant quotations presented to illustrate the themes/findings?
		Yes
		Was each quotation identified?
		No**, they were not identified in order to preserve confidentiality**
30.	Data and findings consistent	Was there consistency between the data presented and the findings?
		Yes
31.	Clarity of major themes	Were major themes clearly presented in the findings?
		Yes
32.	Clarity of minor themes	Is there a description of diverse cases or discussion of minor themes?
		Yes

PGDRM = Post Graduate Diploma in Rural Management; MBA = Master’s in Business Administration; MSW = Masters of Social Work; MA = Master’s in Arts; PhD = Doctor of Philosophy; MD = Doctor of Medicine; IIHMR = Indian Institute of Health Management Research.

## Appendix 2 Sampling matrix

**Table:**

Demographic characteristics	
Age	Less than 45 years/45 years and above
Gender	Male/female
Education	Less than secondary/above secondary
**Service delivery**	
Type of practice	Allopathic/homoeopathic
Average monthly patients	Less than 760/760 and above

### Sample selection

A total of 35 participants were selected purposively by the Maximum Variation Principle on seven criteria–demographic characteristics (age, gender and education) and service delivery (type of practice and average monthly patients) from two geographic locations (deltaic and non-deltaic) of Indian Sundarbans. Based on these seven criteria we created 23 categories; from each category we selected one or two IHPs depending on the proximity to a health centre.

### Sampling categories

**Table:**

Categories	No. of IHP selected based on proximity to a health centre
C1- Less than 45 years/male/less than 760/secondary and above/allopathic	2
C2 - Less than 45 years/less than 760/above secondary/homoeopathic	2
C3 - Less than 45 years/760 and above/above secondary/homoeopathic	2
C4 - Less than 45 years/760 and above/ above secondary/allopathic	2
C5 - 45 and above/less than 760/above secondary/allopathic	2
C6 - 45 and above/less than 760/above secondary/homoeopathic	2
C7 - 45 and above/760 and above/above secondary/allopathic	2
C8 - Less than 45 years/male/less than 760/above secondary/ allopathic	2
C9 - Less than 45 years/male/less than 760/above secondary/ homoeopathic	2
C10 - Less than 45 years/male/less than 760/above secondary/gynaecologist	1
C11- Less than 45 years/male/760 and above/above secondary/skin	1
C12 - Less than 45 years/male/760 and above/above secondary/ allopathic	2
C13 - Less than 45 years/female/less than 760/less than secondary/ homoeopathic	2
C14 - 45 years and above/male/less than 760/above secondary/ allopathic	2
C15 - 45 years and above/male/less than 760/above secondary/ homoeopathic	1
C 16 - 45 and above years/ male/ above 760/ above secondary/ allopathic	2
C17 – 45 years and above/male/above 760/above secondary/ homoeopathic	1
C18 - Less than 45 years/ male/less than 760/above secondary/ allopathic	2
C19 - Less than 45 years/male/less than 760/above secondary/ homoeopathic	1
C20 - Less than 45 years/male/760 above/above secondary/ homoeopathic	1
C21- 45 years and above/male/less than 760/above secondary/ allopathic/secondary/allopathic	2
C22 - 45 years and above/male/less than 760/above secondary/ homoeopathic	2
C23 - 45 years and above/male/760 above/above secondary/ allopathic	1
